# The Person Behind the Crime: Life Stories of Women and Men Who Committed Sexual Crimes

**DOI:** 10.1177/10790632261454292

**Published:** 2026-05-20

**Authors:** Ana Rita Conde, Ana Ferreira, Olga Cunha, Judite Peixoto, Marta Sousa

**Affiliations:** 1 Hei-Lab-Digital Human-Environment Interaction Lab, Porto University Center, Faculty of Psychology and Education, 386292 Lusófona University, Porto, Portugal; 2 Porto University Center, Faculty of Psychology and Education, 386292 Lusófona University, Porto, Portugal; 3 Psychology Research Center (CIPsi), School of Psychology, University of Minho, Braga, Portugal; 4 Hei-Lab-Digital Human-Environment Interaction Lab, Lisbon University Center, EPCV-School of Psychology and Life Sciences, 386292 Lusófona University, Lisbon, Portugal

**Keywords:** sexual crimes, qualitative study, gender, narrative analysis, life stories

## Abstract

Research on individuals convicted of sexual crimes has focused mainly on risk factors and recidivism. Less attention has been given to how these individuals construct meaning around their life trajectories. This qualitative study explores and compares the life narratives of men and women convicted of sexual crimes. Semi-structured life story interviews were conducted with 11 incarcerated individuals: 4 women and 7 men. Data were analyzed using narrative analysis. Five central narrative themes emerged across participants: family, reclusion, intimate relationships, crime, and substance abuse. Participants described adverse family experiences, relational instability, and ambivalent perceptions of imprisonment. Narratives also revealed a tendency to minimize or avoid direct discussion of sexual offending, while constructing a positive and prosocial self-image oriented toward change and reintegration. Gender differences were observed in identity construction and life trajectories. These findings underscore the importance of narrative identity in understanding individuals convicted of sexual crimes. The results highlight the need for gender-sensitive, narrative-informed assessment and intervention. Integrating individuals’ dominant narratives into rehabilitation may help reconstruct identity, improve responsiveness to treatment, and support more effective reintegration.

## Theoretical Framework

Over the past decades, there has been considerable progress in the investigation of sexual crimes (SC) and, specifically, in research with individuals who committed SC (Lussier et al., 2024; McLeod & Dodd, 2022; Sousa et al., 2024; Vandiver et al., 2016). The research has been dedicated mainly on understanding the underlying causes of SC (Chopin et al., 2023; Grady et al., 2018; Oettingen et al., 2024), identifying offense-specific factors - including characteristics or risk factors associated with sexual offending and recidivism (Moretti et al., 2023; Somma et al., 2020), developing and characterizing typologies of individuals who committed SC (Arbanas et al., 2022; Lim et al., 2021; McLeod & Dodd, 2022), and understanding what distinguishes individuals who committed SCs from those who committed other crimes (van der Put et al., 2020; Vandiver et al., 2016). Most studies are quantitative, and their findings vary depending on the theoretical framework used to analyze individuals convicted of SC. That is, in general, different explanations are linked to a variety of theories of crime and SC. Thus, several studies have sought to analyze intra-individual characteristics, such as personality traits (e.g., Sigler, 2017; Sousa, Cunha, et al., 2025), thinking styles (Eberhaut et al., 2023; Marshall et al., 2009), sexual activation (Babchishin et al., 2017), and biological predispositions (Ward & Beech, 2006); others highlight aspects related to social learning and social factors (Akers, 2009), developmental features (Smalbone & Cale, 2015), or advocate an integrative approach (e.g., Seto, 2019; Ward & Beech, 2016).

Statistics and prevalence data indicate that SC are more frequently perpetrated by men against women (World Health Organization [WHO], 2025); nevertheless, numerous studies have been shown that women also commit this type of crime, a reality that cannot be overlooked (Freeman & Sandler, 2008; Sousa et al., 2024). A meta-analysis conducted by Cortoni et al. (2017) found that, according to official statistics, prevalence rates among women who committed SC range from 0.4 and 6.8%. However, victimization surveys report higher estimates, with prevalence rates reaching 3.1% and increasing to as much as 24.4% for crimes committed by women. Moreover, when addressing crimes such as child sexual abuse (CSA), a meta-analytical review found that only about 2% of cases perpetrated by women are officially reported. In contrast, victimization surveys suggest that the prevalence of women who have committed CSA is approximately six times higher than that indicated by official data (Cortoni et al., 2017). This underreporting may occur due to several factors, including the social construction of women as caregivers and their predominant association with the image of the victim rather than of the person who committed a crime (Tozdan et al., 2019). Research indicates that women, like men, also engage in a wide range of abusive sexual behavior (Cortoni & Stefanov, 2020), whether against child victims (Augarde & Rydon-Grange, 2022; McLeod, 2015), adult victims (Fisher & Pina, 2013), or against male and female victims (Augarde & Rydon-Grange, 2022; Cortoni & Stefanov, 2020). Currently, it is recognized that women perpetrate a significant proportion of sexual offences (Cortoni et al., 2017; McLeod & Dodd, 2022), and there has been an increasing interest in the scientific community in the study of women who commit SC.

Significant efforts have been made to analyze and compare female and male individuals who committed SC (Augarde & Rydon-Grange, 2022; Comartin et al., 2018; Williams & Bierie, 2015), to seek risk factors. However, the literature and research are not consistent. There is a consensus on the existence of common risk factors, such as a history of criminal behavior, delinquent peers, employment problems, and family relationships (Freeman & Sandler, 2008). Still, some studies suggest specificities related to women, namely higher rates of mental health problems (e.g., personality disorders, substance use disorders, and mood disorders), a greater history of childhood victimization (e.g., sexual abuse), and adverse experiences in adulthood (e.g., being a victim of an intimate partner) (Augarde & Rydon-Grange, 2022; Comartin et al., 2018; Gillespie et al., 2015; Miller & Marshall, 2019). On the other hand, some authors have identified different patterns of sexual perpetration in women (e.g., earlier age at perpetration, repeated abuse of the same victim, younger victims, existence of a co-offender), but suggest that both men and women share the same risk factors (e.g., victimization, sexual abuse, childhood adversity) (e.g., Comartin et al., 2018).

Despite the growing number of studies that seek to understand the differences and similarities between male and female individuals who committed sex offenses, as far as we know, none of them uses a qualitative methodology. Some qualitative studies seek to understand the perceptions and experiences of male (Brennan et al., 2018; Gannon et al., 2008; Smith, 2022) and female individuals who committed SC (Harrati et al., 2018; Klein et al., 2014) separately, but there are still no studies with a comparative analysis. Also, most of the qualitative studies with male individuals who committed crimes analyze their sexual fantasies during the crime (Gee et al., 2004), the therapeutic process and impact (Blagden et al., 2023; Lindegren, 2025; Sousa, Cunha, et al., 2025), post-traumatic growth (Vanhooren et al., 2018), emotional responses to sexual crime (Brennan et al., 2018), experience of stigmatization (Ricciardelli & Moir, 2013; Sandbukt, 2021), life trajectories (Eloir et al., 2020; Naidoo, & Van Hout, 2021), and narratives and identity (Mullins & Kirkwood, 2024; Sandbukt, 2025). Regarding qualitative studies with women who committed crimes, there are fewer that explore their traumatic experiences (Harrati et al., 2018), motivations and cognitions (Beech et al., 2009), life events, prison experience, and emotions (Klein et al., 2014). Steely and Bensel (2019), for example, analyzed the narratives of female individuals who committed crimes in the educational context and identified several categories of offending teachers, such as predatory, sexually friendly, and emotionally dependent.

To our knowledge, no qualitative studies have examined and directly compared men and women who committed SC to analyze their life trajectories, their constructions of personal identity and life narratives, and how they integrate past, present, and future experiences. As McAdams (2008) argues, individuals construct meaning to various experiences throughout their lives by organizing them into a story, and this life story provides a sense of coherence, continuity, meaning, and purpose. However, disruptive or adverse life experiences, such as being convicted of a crime and imprisonment, may undermine the coherence of these life stories and challenge an individual’s sense of identity (van Ginneken, 2014).

Gender, a social construction resulting from the complex interaction among biological sex, developmental factors, and social, cultural, and historical meanings (Fivush & Grysman, 2022), influences how people create meanings about themselves and their lives. Gender constructions convey expectations about behavior, roles, attitudes, and even the image associated with men and women, with what some authors call the “master narrative of gender” (Breen et al., 2017; Fivush & Grysman, 2022; McLean & Breen, 2016) that explicitly creates gendered expectations about people’s lives and their way of being and behaving. Deviations from these master narratives are negatively connoted and subject to severe criticism or sanctions, especially in the case of women, either by others and by society in general or by the woman herself (Miller, 2005). The condition of being a woman who committed crimes - particularly SC - challenges dominant cultural constructions of femininity and womanhood, which are commonly associated with traits such as care, protection, passivity, and non-aggression (Crosland et al., 2025; Sandman, 2022).

In general, SC carries a strong negative social connotation (Sandbukt, 2021; Schultz, 2014), resulting in pronounced social stigmatization and public alarm directed toward individuals who committed such offenses (Cubellis et al., 2019). Considering the different gender constructions, it is therefore important to understand how women and men who committed SC integrate these experiences into their life stories and personal identities.

## Method

### Narrative Approach

Authors from constructivist and social constructionist perspectives highlight the narrative nature of human beings: that is, events and situations gain individual meaning when organized into a narrative or story (McAdams, 2008; Morgan, 2002). People engage in the activity of telling a story about what happened to them, about the different events over time, organizing them chronologically, and according to the meaning (McAdams, 2008). By telling one’s story, one may give meaning to experiences lived throughout life, building one’s own vision of the self and personal identity (McAdams, 2008; Morgan, 2002). Thus, the narrative approach seeks to understand how people create their life story and personal identity, and how they integrate their past (reconstructed), present (perceived), and future (anticipated) selves (McAdams, 2008). Life stories and narratives provide a sense of coherence, meaning, and purpose. Nevertheless, some experiential situations may represent abrupt changes in the life cycle and pose challenges to one’s identity (Mansfield et al., 2015; Pals, 2006).

Individuals who committed SC are no exception, so it is relevant to consider their narratives to understand their involvement in sexual offences and how they deal with the criminal consequences. As Pals & McAdams (2004) suggest, attributing meaning is an active process of re-evaluating an event; thus, it is important to identify the meanings constructed by individuals who committed SC, given their situation of reclusion and the reasons that led them to this condition.

The narrative approach contributes to the development of a framework that enables a comprehension of crime applicable to different types of individuals who committed crimes (Ioannou et al., 2015), including women and men who committed SC. This framework also allows a better understanding of the rehabilitation process to minimize recidivism (Ward & Marshall, 2007), with studies indicating that self-narratives can allow the reconstruction of individuals’ identities so that crime is excluded from them. Thus, going beyond behavioral explanations, the narrative approach allows not only to understand how individuals who committed crimes deal with crime but also to understand their process of giving up on crime and/or its recurrence (Presser, 2009).

### Goals

Accordingly, the present study seeks to adopt a narrative approach with individuals who committed SC, with a focus on understanding how women and men in prison convicted of a SC shape and understand their life stories. Given differences in research on women and men who committed crimes, the goal is to compare their life narratives and narratives of themselves as people.

To this end, the following research questions (Q) were formulated:

Q1: Which are the central narrative themes that arise in the life story of individuals who committed SC? What are the key elements, and how are they described? Does the crime that led to their conviction emerge as a theme in their life stories? If so, how?
Q2: How do they describe themselves? How do they build their identity?
Q3: Are there differences between women and men who committed crimes? If so, what are they? How do they emerge? Is it possible to identify a common narrative between women and men, or are they clearly different? How do these narratives relate to SC?

### Participants, Sampling, and Data Collection Procedure

This study included 11 participants convicted of SC, selected according to the following inclusion criteria: (i) being convicted of a SC; (ii) serving an effective prison sentence; (iii) cognitively able to understand and report situations, problems, and events; and (iv) not presenting indicators of active consumption of psychoactive substances. Considering the objectives of this study, the participants’ selection followed an intentional sampling strategy (Flick, 2007), that is, participants who better corresponded to the intended profile according to the inclusion criteria.

The definition of the number of participants in narrative research does not follow statistical criteria, but rather principles of depth, richness, and diversity of life stories (Creswell, 2013; Riessman, 2008; Staller, 2021). Narrative analysis aims at a deeper understanding of life trajectories and the meanings attributed to experiences; therefore, small purposive samples are recommended to allow for a detailed and contextualized narrative analysis.

In the present study, 11 individuals convicted of sexual crimes participated, four women and seven men (see Table 1). This distribution stems, firstly, from the reality of the prison population in Portugal, where the number of women convicted of this type of crime is very small. Thus, the number of participants reflects the diversity and proportionality existing in the prison context.

**Table 1.** Participants

Participants	Gender	Age	Type of crime
Participant 1	Female	41	Child sexual abuse
Participant 2	Female	36	Child sexual abuse
Participant 3	Female	52	Child sexual abuse
Participant 4	Female	44	Child sexual abuse
Participant 5	Male	31	Sexual offense against women
Participant 6	Male	40	Child sexual abuse
Participant 7	Male	56	Sexual offense against women
Participant 8	Male	30	Both crimes
Participant 9	Male	34	Sexual offense against women
Participant 10	Male	40	Sexual offense against women
Participant 11	Male	35	Sexual offense against women

Additionally, the literature indicates that comparative narrative studies can include 5–15 participants, and it is equally acceptable to include smaller subgroups when these correspond to populations that are difficult to access or less represented (Creswell, 2013; Riessman, 2008).

To collect data, authorization was requested from the General Directorate of Reinsertion and Prison Services. After authorization, contact was established with prison directors to disseminate the information about the study and to schedule in-person data collection. Each prison director was previously informed of the inclusion criteria, and prison staff identified potential participants accordingly. Based on this selection, each participant was approached individually to invite them to participate in the study. For those who expressed interest, informed consent was obtained after explaining the study’s general objective, procedures, the voluntary nature of participation, and the anonymity of participants. Of the potential participants (15), four declined to participate (three men and one woman).

The interview was administered individually, audio-recorded, and fully transcribed to ensure accuracy. It should be noted that to identify the type of SC committed, the information was provided by prison services.

The study was submitted to the Institutional Ethics Committee, and data collection began only after its approval, ensuring that all procedures complied with the research committee’s ethical standards and the 1964 Declaration of Helsinki and its subsequent amendments.

### Instruments

McAdams Life Story Interview (McAdams, 2008) was used to obtain participants’ life story reports and to obtain adequate data for narrative analysis. It is a semi-structured interview designed to explore life trajectories, the meanings attributed to personal experiences, and the factors that led to the commission of a crime. The script identifies main life chapters, describes key scenes (such as highs, lows, turning points, and significant childhood and adult memories), explores future expectations and life projects, and reviews life challenges (like losses, failures, and adverse experiences). It also explores values, beliefs, and personal ideology. This narrative interview collects in-depth information on personal development, risk and protective factors, decision-making, accountability, and perspectives on change and social reintegration. It helps develop a more comprehensive understanding of the life trajectories of men and women convicted of SC.

### Data Analysis

Narrative analysis was used as the most appropriate strategy given the study’s nature, objectives, underlying theoretical approach, and data collection technique (the life story interview) (Howitt, 2016; Murray, 2008). Given the variability of procedures found in the literature on narrative analysis, Howitt’s procedures (2016) were chosen because they were presented in a more organized and systematic way. On the other hand, these procedures also employ narrative analysis grounded in narrative psychology, drawing on concepts such as self and identity arising from the narrative (Murray, 2008). The NVivo 10 data analysis software (QSR International, 2012) allowed an adequate analysis organization.

We organized the transcripts into meaningful segments to guide our analysis and coding. Following transcription, the interviews were read repeatedly to gain deeper familiarity with the data, allowing for the identification of narrative patterns, meanings, and recurring elements in participants’ life stories.

First, we identified central themes in each life story. We analyzed the narrative content to find patterns, recurring meanings, and key events. Themes emerged inductively from the narratives and were organized by importance and frequency.

In a second phase, other narrative elements were identified, namely the narrative tone, the images, and the metaphors used. The narrative tone was analyzed considering both the content of the reported experiences and the way they were narrated, allowing us to understand the participants’ subjective perspective on their life trajectories. Narrative images and metaphors are symbolic representations that deepen our understanding of the meanings attributed to personal experiences and trajectories, reflecting how participants conceptualize their life trajectories and personal identity.

Finally, a holistic analysis of the narratives was carried out, integrating the identified elements (themes, narrative tone, and images) into a coherent interpretation, allowing the identification of the participants’ dominant narratives and articulating these themes with the objectives of the study.

To ensure the credibility and rigor of the narrative analysis, strategies such as repeated readings, in-depth analysis, and grounding interpretations in participants’ narratives were employed, ensuring consistency and fidelity to the original data (Andrews, 2021; Crossley, 2000; Riessman, 2008).

We took a holistic approach, treating each interview as a complete unit and preserving story coherence (Crossley, 2000; Murray, 2015). We also compared interviews to identify common patterns, differences, and gender variations, strengthening credibility through triangulation (Kyngäs et al., 2020; Pino Gavidia & Adu, 2022). We used illustrative excerpts from the interviews to support our interpretations and ensure transparency (Andrews, 2021; Riessman, 2008). Throughout, the researcher remained reflexive, recognizing the co-constructed nature of narrative analysis (Pino Gavidia & Adu, 2022).

The researchers have previous academic and professional experience working with justice-involved populations, which informed their interest in understanding the life narratives of individuals convicted of sexual crimes. This background may have facilitated rapport during interviews, while also shaping sensitivity to issues related to stigma, identity reconstruction, and social reintegration. To enhance reflexivity and reduce interpretative bias, the researchers engaged in regular discussions throughout the analytical process, comparing interpretations and assuring that findings were grounded in participants’ narratives. This process is intended to strengthen credibility and transparency in the narrative analysis.

## Results and Discussion

For clarity and organizational coherence, the results and discussion are structured according to each research question.

### What Are the Central Narrative Themes That Arise in the Life Story of Individuals Who Committed SC? What are the Key Elements, and How Are They Described? Does the Crime That Led to Their Conviction Emerge As a Theme In Their Life Stories? If So, How?

Analysis of participants’ life stories revealed five themes: family, reclusion, intimate relationships, crime, and substance abuse (see Table 2).

**Table 2.** Main Themes Addressed by the Participants

Themes	Number of participants	Number of references
Family	11	181
Reclusion	11	25
Crime	9	47
Intimate relationships	7	37
Substance abuse	5	15

### Family

Both men and women are mostly influenced by their families throughout their lives. In this context, particular emphasis is placed on negative experiences, especially those involving loss and death.

P2 - I lost my father very early… it was just me, my mother, and my sister.

P7 – It was the death of my uncle, but mainly from an uncle of mine who killed himself, and that was remarkable…

Participants also reported experiences of mistreatment and neglect during childhood.

P1 - I suffered a lot, and I saw my mother suffer a lot because of my father; she suffered from mistreatment, domestic violence, and that marked me.

P6 - My father was an alcoholic; I was begging to be able to eat and would have slept anywhere.

However, participants also referred to positive family experiences, namely moments of affection and happiness within their family of origin, as well as the birth of their children.

The identification of adverse life and family experiences in participants’ narratives is in line with the existing literature and studies, which indicates that histories of abuse and neglect, loss of parental figures, inappropriate parenting practices, and abandonment are common among individuals who have committed SC (e.g., Harrati et al., 2018; Sandvik et al., 2017). Such traumatic and adverse experiences - including those of mistreatment and the loss reported by participants - may lead to difficulties in emotional regulation, interpersonal problems, and the development of cognitive distortions, which in turn may sustain sexual offending and its perceived legitimacy (Harrati et al., 2018; Sandvik et al., 2017; Sousa, Gonçalves, et al., 2025; Ward & Beech, 2016). Indeed, a recent qualitative study revealed that participants reported a link between the adverse experiences they had endured and the perpetration of SC. Although there is no direct association, the participants reported that these experiences have influenced areas of functioning such as their interpersonal patterns and cognitive schemas (Grady et al., 2022).

### Reclusion

Reclusion, as participants’ current life context, emerged as another central theme. It was predominantly described in negative terms (P5 – *I came to prison, my life stopped here;* P2 – *I’m here, which was something I thought would never happen to me, it is very bad to be here*); however, some participants also conceptualized incarceration as an opportunity for reflection and learning (P7 – *Having come here to stop, changed my way of seeing things and it was a life lesson*; P1- *It’s being here in jail, it made me change a lot the way of being, a person can reflect on things, makes us grow a lot*). This ambivalence corroborates previous studies on individuals who committed SC, which similarly highlight the coexistence of negative and constructive perceptions of imprisonment (Cooper & Holgersen, 2016). These findings may also reflect how individuals perceive both the crime they committed and their experience within the prison environment. Specifically, the presence of beliefs that minimize the seriousness of the offense may lead to a negative appraisal of the imposed sentence, which is viewed as unnecessary. Moreover, negative perceptions of the prison system may also be linked to experiences within this setting, as individuals convicted of sexual offenses often face greater adjustment difficulties, being more frequently victimized by other inmates and staff (e.g., van den Berg et al., 2018; Wuyts et al., 2023).

### Intimate Relationships

Narratives concerning intimate relationships were likewise marked by ambiguity: on the one hand, participants emphasized union, affection, and happiness (P5 - *I got married, it was one of the good moments, it was almost 7 years of marriage*; P4 - *My marriage was a happy marriage, I stayed with my husband many years*); on the other hand, they described multiple difficulties (P5 - *At the wedding, I was a little disturbed and then I started with drugs*; P1 - *At my wedding too I made a mistake when I was with my ex-husband, I made mistakes as a wife, as a mother*) that ultimately culminated in the rupture of the relationship.

This ambiguity in the conceptualization and experience of adult intimate relationships may reflect difficulties in emotional regulation, as suggested by the literature (e.g., Sandvik et al., 2017), as well as deficits in social and intimacy-related skills (Ward & Beech, 2016). Additionally, prior research indicates that stressors and adverse life circumstances, such as the rupture of an intimate relationship, can also contribute to sexual offending, namely in cases involving the abuse of minors by non-exclusive individuals (Wojcik & Fisher, 2019). Furthermore, these difficulties are considered a central risk factor for sexual reoffending (Seto et al., 2023).

### Crime

Crime emerged as another salient theme in participants’ narratives, primarily to account for their current imprisonment. Participants’ accounts largely emphasized expressions of regret (P7 - *Repentance at what I did*; P10 - *My biggest regret is the crime*), while rarely making explicit reference to the sexual offence itself. Instead, they tended to minimize, deny, or avoid the SC (P1 – *It touched me a lot because of the situation that led me to be here in jail, because it was the other person who abused my daughter*), often framing their incarceration as resulting from other types of crime (P6 - *I did not expect to be arrested because in the searches made by the police, no drugs were ever found, I was condemned to the conviction, the drug never existed*) or displacing responsibility onto others for their involvement in criminal activities (P6 - *I was condemned to the conviction by my friends, by friends who got into drugs*; P2 - *If I did not think about others at the time and had thought about me, I would not be here*).

This tendency to deny or reframe the sexual offence by invoking other forms of crime may be related to the particularly negative social connotations associated with sexual offending (Ricciardelli et al., 2025; Sandbukt, 2021) and may function as a strategy to avoid or mitigate the strong social stigmatization to which individuals convicted of sexual offences are subjected (Cubellis et al., 2019).

### Substance Abuse

Another central theme is substance/drug abuse, which participants described both as a source of personal problems (P1 - *Having been involved with drugs, this was a failure for me; it was the beginning of the end*), and in some cases, as something they took pride in overcoming (P6 - *Getting rid of drugs and alcohol was difficult, but I did it*). Substance use is a common issue among individuals who have committed crimes in general, including sexual offences (Stinson & Becker, 2011), and is considered a risk factor for the perpetration of violent behaviors, particularly sexual violence (Briken et al., 2020; Kirk-Provencher et al., 2021).

### How Do They Describe Themselves? How Do They Build Their Identity?

Throughout their descriptions of experiences and life events, several factors emerge as shaping their identity development, including the ways they characterize themselves, how they project the future, and the beliefs and values they hold (see Table 3).

**Table 3.** Description of Themselves/Others, Projection Into the Future, Beliefs/Values

​	Number of participants	Number of references
Self-characterization	10	31
Resilient, fighters, determined	10	19
Sociable, affable, caregivers	6	13
In relation to others	10	27
Vulnerable to bad company/circumstances	9	19
Projection into the future	11	33
Getting a job/having a profession	10	15
Establish/recover family	5	8
Evolve as a person	3	4
Beliefs and values	11	35
Religious faith	11	20
Honesty/friendship/sincerity	4	4
Life as a value	3	3
Family as a value	7	10

Overall, despite their crime and incarceration, participants’ narratives are organized in ways that construct a positive self-image, portraying themselves as resilient, and determined in facing adversity (P2 - *Not giving up because I was never a person to give up, I will always fight! I’ve been through so much in life and never gave up*; P1 - *It made me feel more mature, I grew a lot, it made me grow a lot*), and, at the same time, friendly and sociable (P5 - *I am family, I am union, I am a good boy*; P6 - *I think people trust me and respect me*; P4 - *I was always held in high esteem by everyone*).

To further reinforce this positive self-image, they also use descriptions of “others” as resources to bolster it. They describe themselves in their relationship with others as vulnerable to negative friendships or adverse circumstances (P6 - *This was a delay in my life; it was all due to bad friendships. With this situation that happened in my life, I must consider the choice of friends*; P11 - *Bad friends also didn’t help*; P9 - *My biggest regret was giving confidence to certain people, otherwise I wouldn’t be here today*) to justify their current situation, including their conviction for a crime.

Another dimension of identity was how they project themselves into the future. The development of a positive self-image allows them to project themselves into the future in a prosocial way or in accordance with current social rules, namely finding a job and having a profession (P7 - *Returning to work, helping my children and my grandchildren, go back to the profession that I had and that I loved*; P10 - *I want to continue the chapter that was stuck, continue with the life I had outside, mainly at work level*), start a family (P7 - *Live with the family, create my own family, own a house, a car, be happy*), and evolve as a person (P6 - *Organize my life, work, respect people, and be a better person*). Another dimension that their narratives convey was the “moral” dimension of beliefs and values. It should be noted that all of them refer to religious/spiritual faith for a sense of security and protection (P2 - *I believe that there is God, and that the Bible, which is the word of God, has good advice, and if we follow it, we will be successful*). In terms of values, they emphasized friendship/honesty/sincerity (P5 - *…sincerity and friendship complement each other, with both, I think everything goes well*), which allows them to sustain their positive self-image and differentiate themselves from the “bad companies” they refer to throughout their history. Finally, they take life as another relevant value (P2 - *The most important value in life… life itself is the most important*), as well as the family (P6 - *My mother's love because she is my friend. For me, my mother is everything*), also contributing to the reinforcement of a positive and pro-social self-image.

As can be seen, the participants seek to build an identity that excludes criminality, i.e., to reconstruct a prosocial identity. If, on the one hand, this can be understood as an attempt to deny a crime, to seek social desirability, or as a lack of insight into the crime, on the other hand, it can be understood as an opportunity to change their life trajectories. The identity components of their accounts can open opportunities for personal development and a positive reconstruction of their own identity (van Ginneken, 2014), expressing their potential as a foundation for desistance and moral growth. This is in line with the study by Presser (2004), who identified in the report of the individuals who committed crimes the search for their “moral self” to distance themselves from the problematic social construction of violent offences. The reconstruction of a non-offending identity narrative can act as a catalyst for self-development and positive change (Lindegren, 2025). This perspective aligns with transformative, strengths-based rehabilitation models, such as the Good Lives Model, which emphasize moral identity, purpose, and social connectedness as key mechanisms for change (Ward et al., 2025; Youssef, 2025).

Furthermore, the religious/spiritual faith that emerged among all participants aligns with studies indicating that faith serves as a form of identity transformation and a facilitator of abandoning crime (Plimley, 2024).

### Are There Differences Between Women and Men Who Perpetrated SC? If So, What are They? How Do They Emerge? Or Is It Possible to Identify a Common Narrative Between Women and Men, or Are They Clearly Different? How Do These Narratives Relate to SC?

Distinctive patterns were identified in the narratives of male and female individuals who committed SC. Men and women referred to the same narrative themes, but differences emerged regarding substance abuse. Most male individuals who committed crimes described a life trajectory closely linked to substance use, framing it as the primary source or starting point of their problems and criminal trajectory.

This finding aligns with several studies involving forensic populations, which indicates that a history of substance abuse is a risk factor for offending and is more commonly observed in men (de Vogel et al., 2022). Men are also more frequently diagnosed with substance dependence than women (de Vogel et al., 2022). Research involving individuals who committed SC shows that a significant proportion present with a substance-related disorder. Alcohol abuse is more prevalent among individuals who committed SC than among individuals who committed non-sexual violent crimes, and drug use appears to be more common among individuals who committed SC against women than those who committed child abuse (Briken et al., 2020). The association between substance use and sexual offending is complex; while substance use may act as a disinhibitory and contribute to the commission of SC, it is generally not considered the primary cause.

Another differentiating aspect concerns identity development. Male individuals primarily focus on self-characterization and on descriptions of others to reinforce a positive self-image. In this context, men emphasize their sociable and affable qualities and highlight their vulnerability to negative friendships and adverse circumstances, often linked to their trajectory of substance use. They also place considerable value on relational components, namely friendship, sincerity, and family. These findings contrast with studies of other types of individuals who committed crimes (non-sexual crimes), which indicate that women who committed crimes tend to construct more relational narrative, while male narratives are more oriented toward social status (Herrschaft et al., 2009).

In contrast, women in the present study most frequently described themselves as resilient, determined, and “fighter-like”. It could be said that women convey the image of “warriors”, whereas men present themselves as “affable” individuals who, due to their sociable nature, have become more vulnerable or victims of “bad friendships”. Qualitative research supports this distinction, showing that female individuals who committed crimes often employ narratives emphasizing resilience in the face of adversity (Ciesla et al., 2019), while men more frequently frame their criminal behavior through external or relational justifications (Hamilton & Sanchez, 2019).

Through the articulation of narrative themes, identity components, narrative tone, and imagery, a shared narrative pattern can be identified among both female and male individuals who committed crimes: a narrative of adversity accompanied by dissociation from or denial of the sexual offence. Across participants’ life stories, there are no explicit references to SC; instead, narratives consistently revolve around adverse and negative experiences. These accounts appear to function as explanatory frameworks for their deviant or criminal life trajectories, while avoiding direct engagement with the sexual offence itself (P4 - *I was always a person who felt inferior at school because I was poor and others had things that I didn’t have; we were a big family and I just wanted to hurt them, I really needed to hurt them very badly…*).

However, two distinct narratives also emerged from this overarching pattern: the narrative of resilient adversity and the narrative of suffering or victimizing adversity, both of which serve to maintain a positive self-image. The narrative of resilient adversity depicts a life trajectory marked by difficulties and obstacles - including crime and imprisonment - that are framed as contributing to personal growth, learning, and enhanced resilience by fostering perseverance. This narrative is associated with the image of the “warrior” or “fighter” and adopts a more optimistic tone, emphasizing personal agency and the capacity to shape one’s future.

P2 - The fighting woman that I am is for him (son) (…) “the warrior”… I’ve been through so much in life and never gave up.

P1 - Because life is a big fight, we must fight to have it, fight for our family, to achieve our goals, we cannot throw our arms down.

The narrative of victimizing adversity or suffering is likewise marked by negative experiences, difficulties, and obstacles. However, it emphasizes the adverse effects of these experiences and the individual’s vulnerabilities. This narrative is associated with the image of a victim of life’s circumstances and is conveyed in a predominantly suffering-oriented tone, limiting the perceived capacity for personal agency and future action.

P5 - Failure is to have fought so hard and not succeeded.

P3 - Coming here is a curse! I don’t wish anyone this. Not even my biggest enemy deserves to be here.

Both male and female participants follow a common narrative structure, focusing on adversity while frequently exhibiting dissociation from or denial of sexual offences. This narrative functions to protect their identity. Participants account for their life trajectories primarily in terms of structural and relational factors, such as poverty, social exclusion, and family dysfunction, while omitting sexual offenses from their life stories to maintain a positive self-image and separate their identity from their criminal behavior. For example, P4 shifts the focus from the offence to feelings of humiliation and anger, highlighting personal injury and offering a rationale for deviant behavior without directly acknowledging the SC. Within these narratives, two distinct identity trajectories emerge. The first, the narrative of resilient adversity, highlights agency, perseverance, and a self-image as a “fighter”. The second, the narrative of suffering adversity, is characterized by victimhood, pessimism, and limited hope. Both narrative types serve to protect identity by keeping sexual offences excluded from participants’ personal histories.

## Conclusion

The present study aimed to explore how women and men imprisoned for sexual crimes construct and make sense of their life stories. The findings of the present study highlighted two key themes in participants’ narratives: (1) trajectories marked by childhood adversity that evolved into narratives of adversity accompanied by dissociation and/or denial of the offense; and (2) the use of contrasting metaphors, namely the ‘warrior’ metaphor, associated with narratives of resilient adversity, and the ‘affable victim’ metaphor, linked to narratives of suffering or victimized adversity.

### From Experiences of Adversity in Childhood to a Narrative of Adversity Accompanied by Dissociation or Denial of Crime

Individuals who committed SC present a narrative that focuses on adverse early family experiences, namely a history of losses, abuse, and family neglect. The literature indicates that childhood experiences of loss, rejection, abuse, mistreatment, and neglect can contribute to sexual aggression, through the attachment difficulties, deficits in intimacy skills, emotional dysregulation, and the development of hostile attitudes (Sousa, Cunha, et al., 2025). These findings are not surprising when considering that, if such experiences remain unprocessed, they may lead to an excessive focus on personal adversity, driven by a motivation to have one’s victimhood acknowledged and met with empathy (Gabay et al., 2020; Twali et al., 2020). In this regard, trauma-focused and trauma-informed interventions should be considered not as a means of minimizing the offenses committed, but as an additional piece of the puzzle (Grady et al., 2022). Such interventions should constitute first-line approaches for individuals with unresolved childhood adversities, followed by interventions targeting risk factors associated with the perpetration of sexual crimes (Grady et al., 2022). Indeed, trauma-focused approaches have demonstrated positive effects in reducing post-traumatic stress symptoms within this population (Sousa et al., 2024; 2025b). The aim is to reconceptualize adverse or negative experiences so that they are no longer used to legitimize a deviant or criminal trajectory, thereby supporting the reconstruction of a life path free from the enduring effects of adversity and away from crime (Sousa, Gonçalves, et al., 2025).

Notably, none of the participants acknowledged committing SC; instead, they cited other types of crime to justify their incarceration or avoided addressing the cause of their confinement altogether. Women, in particular, frequently attributed criminal agency to third parties (e.g., partners or accomplices). Research indicates that a distinctive feature of women is involvement in abuse alongside an accomplice or partner (Wijkman et al., 2010). Accordingly, intervention plans must address this specificity, emphasizing the importance of taking responsibility for the offence, even in cases where the crime was “coerced” or “motivated” by another individual.

In the case of men, attributing their incarceration to other types of crime may be related to the social shame and stigmatization associated with sexual offences. However, the literature indicates that individuals who have committed crimes and feel shame and avoid acknowledging their culpability for such crimes are more likely to continue blaming victims and show greater resistance to intervention (Marshall et al., 2005). Therefore, intervention plans should aim to deconstruct the social stigma associated with sexual offending and promote the development of a responsible self-narrative that is not dominated by shame or the crime itself.

### The “Warrior” Metaphor and the Narrative of Resilient Adversity Versus the “Affable Victim” Metaphor and the Narrative of Suffering or Victimizing Adversity

Women tend to construct narratives emphasizing struggle and resilience when facing life’s adversities, including incarceration, portraying themselves as “warriors” who refuse to be overwhelmed by difficulties or to give up in the face of obstacles – resilient in the face of adversity. In contrast, men paradoxically present narratives characterized by heightened vulnerability, highlighting their affable and credulous traits and depicting themselves as victims of others or of adverse circumstances. Their accounts place greater emphasis on the negative effects of adversity and personal vulnerability, reflecting a narrative of suffering or victimizing adversity.

These distinct narratives have important implications for intervention outcomes and recidivism risk. The narrative characterized by a more optimistic tone conveys greater personal agency and orientation toward the future, whereas the suffering or victimizing narrative diminishes personal agency and may hinder involvement and motivation in processes of change. As Wright et al. (2018) observed, an important factor in promoting behavioral change and desistance from crime is the experience of agency, namely the capacity to identify goals and perceive a sense of control over one’s future. Thus, parallels can be drawn between interventions with victims and with individuals who committed crimes, as both require addressing experiences of adversity and promoting a sense of agency. Accordingly, interventions should focus on strengthening personal agency by developing planning skills, supporting the implementation of actions directed toward prosocial goals, and enhancing individuals’ perceived ability to exert control over their lives rather than being determined by adverse circumstances.

## Limitations

This study presents some limitations. First, the accuracy of participants’ accounts depends on the reliability of their memory as well as on their willingness to disclose personal information during the interviews. Second, the sample size is limited, which highlights the need for further interviews. Notably, the small number of women who have committed sexual offences reflects the reduced number of convictions for such crimes in Portugal.

## Data Availability

The data that support the findings of this study are available from the corresponding author, upon reasonable request.*
